# The longitudinal effects of neonatal anthropometry on attention problems in males and females

**DOI:** 10.1002/jcv2.12256

**Published:** 2024-07-29

**Authors:** Lars Meinertz Byg, Carol Wang, Jonathan J. Hirst, Roger Smith, Craig Pennell

**Affiliations:** ^1^ School of Medicine and Public Health University of Newcastle Newcastle NSW Australia; ^2^ Mothers and Babies Research Centre Hunter Medical Research Institute Newcastle NSW Australia; ^3^ School of Biomedical Sciences and Pharmacy University of Newcastle Newcastle NSW Australia; ^4^ John Hunter Hospital Endocrinology Department Newcastle NSW Australia

**Keywords:** attention, birth weight, childhood behavior checklist, developmental origins of health and disease, head circumference, longitudinal cohort, ponderal index, proportion of optimal birthweight

## Abstract

**Background:**

The longitudinal impact of fetal growth on attention problems in males and females is unclear. This study aims to evaluate the impact of fetal growth assessed by neonatal anthropometry throughout childhood and adolescence in males and females separately.

**Methods:**

We compared neonatal anthropometry (birth weight (BW), head circumference (HC), proportion of optimal birthweight (POBW)) and asymmetry (head‐to‐abdominal circumference ratio (HC/AC) and ponderal index (PI)) at birth with parental assessment of the child behavior checklist attention‐problem syndrome (CBCL‐AP) raw score measured at ages five, eight, 10, 14 and 17. We used univariable and multivariable linear mixed‐effects modeling. Sensitivity analyses included excluding pre‐term births, teacher ratings and treating the CBCL‐AP as an ordinal variable.

**Results:**

In males, a 1‐SD lower BW, increased CBCL‐AP by 0.234 (95%CI [−0.422, −0.0497]). In males, a 1‐SD lower HC increased CBCL‐AP by 0.316 (95%CI [0.495, 0.133]). In males, there was a U‐shaped relationship between HC/AC and CBCL‐AP throughout childhood and adolescence; a curvilinear relationship was observed between POBW and CBCL‐AP. In females, a 1 SD lower HC increased CBCL‐AP 0.424 (95%CI [0.726, 0.133]), but every increased year of age reduced the effect by 0.027 (95% *CI*: 0.006–0.05). In females, there was no clear relationship between BW, POBW or HC/AC and CBCL‐AP. In males and females, PI was not significantly associated with CBCL‐AP. The exclusion of pre‐term births and analysis of teacher‐rated attention problems was consistent with the primary results.

**Conclusions:**

Using a longitudinal design, our study suggests a male vulnerability to attention problems throughout childhood and adolescence from neonatal anthropometry. The relationships in females appear to be limited to childhood.

## Graphical abstract

Previous studies have demonstrated sex differences in the relationship between neonatal anthropometry and increased attention problems. Our study demonstrates that the nature of this relationship ‐ quadratic versus linear ‐ varies according to the anthropometric measure used and that the impact of increasing age on these effects differ in males and females.

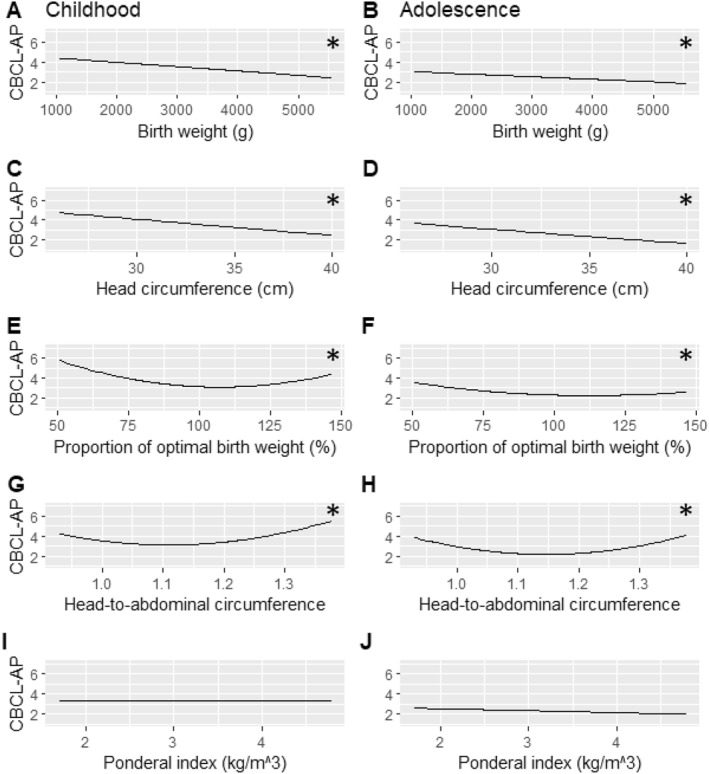

**Key points**

- Lower birth weight and head circumference was associated with more attention problems in males throughout childhood and adolescence.
- Head‐to‐abdominal circumference ratio and the proportion of optimal birth weight had quadratic relationship with attention problems in males throughout childhood and adolescence.
- Lower head circumference was associated with more attention problems in females with a reduction in the effect size as the participant age increased.
- What's relevant.
- These findings suggest lasting psychological impacts of fetal growth primarily in males throughout pre‐adulthood and encourage vigilance for the emergence of attention disorders in this vulnerable population.

## INTRODUCTION

Attention problems are a global issue associated with adverse health and economic outcomes throughout life (Daley et al., 2019; Dalsgaard S, 2015). Attention‐deficit hyperactivity disorder (ADHD), the most studied manifestation of attention problems, affects 85 million individuals (Ferrari A, 2022) and can be present from childhood (Leache et al., 2021). While ADHD is dichotomised through clinical diagnosis, attention problems exist on a continuous spectrum (Coghill & Sonuga‐Barke, 2012; McLennan, 2016) and can be measured using the childhood behavior checklist (CBCL‐AP).

Twin studies and mendelian randomisation provide strong evidence that low birth weight (LBW) increases the diagnosis rate of ADHD (Lim et al., 2018; Orri et al., 2021; Pettersson et al., 2015) in line with the theory of the developmental origin of health and disease (DOHaD) (Geva et al., 2006; Murray et al., 2015). Longitudinal studies suggest a stabile association between LBW and attention problems (Bohnert & Breslau, 2008; Martel et al., 2007; Momany et al., 2018), and some report that these effects stem from a concurrent reduction in head circumference (HC) (Aagaard et al., 2018); however, potential effects may be subject to more complexity, as a limited amount of studies have associated neonatal asymmetry (Lahti et al., 2006) and higher BW with increased CBCL‐AP (van Mil et al., 2015) with more recent studies suggesting potential sex‐differences.

Male sex increases the risk of ADHD diagnosis (Efron et al., 2013), and males display higher symptom severity (Arnett et al., 2015). There have been reports of sex differences in the effects of neonatal anthropometry on CBCL‐AP (Dooley et al., 2022; Momany et al., 2017; Murray et al., 2015) with female vulnerability at age four and male vulnerability around ages 10 and 11. A meta‐analysis showed no effect of sex on the linear association between BW and attention problems, but demonstrated that, use of dichotomous measures of BW and CBCL‐AP influenced estimates, potentially introducing bias; furthermore, previous studies each assessed the sex‐specific effects at single time points.

Attention problems can decrease with age, and the persistence of symptoms from childhood into adulthood is reported to be 40% (Sibley et al., 2016). Recent findings have highlighted the possibility of a second diagnostic category of ADHD, with few symptoms in childhood and the emergence of an ADHD phenotype in adulthood without a clear male predominance (Agnew‐Blais et al., 2016; Caye et al., 2016; Robbers et al., 2011; Terrie E. Moffitt et al., 2015). Consistent with this, ADHD trajectories in childhood and adolescence vary by sex (Murray et al., 2019). We recently demonstrated that the behavioral effects of birthweight in childhood and adolescence are contingent on offspring sex (Lars Meinertz et al., 2023) but the temporal stability, potential quadratic nature and relationship with other measures of neonatal anthropometry remain unexplored.

### Objectives

The aim of this study was to examine the linear and quadratic relationship between fetal growth on attention problems in males and females across childhood and adolescence using repeated measures. We hypothesised that quadratic models would be superior and that increasing age would attenuate associations differently in males and females.

## METHODS

### Study population

We analyzed data from the Raine Study (https://rainestudy.org.au/) (Newnham et al., 1993). The Raine Study is a longitudinal study following mother‐baby dyads recruited at or around 18 weeks gestation (*n* = 2979) through the public antenatal clinic at King Ed‐ward Memorial Hospital and nearby private clinics in Perth, Western Australia, from May 1989 to November 1991. Offspring were followed up throughout childhood with modest attrition of mothers with lower age, education, income and non‐european ancestry (Dontje et al., 2019; White et al., 2017). The Human Research Ethics Committees at the University of Western Australia, King Ed‐ward Memorial Hospital, and Princess Margaret Hospital in Perth, Australia, granted ethics approval for each follow‐up in the study.

### Outcome variables

Using parent reports of CBCL for Ages 4–18 (CBCL/4–18), we derived scores of attention problems at ages five, eight, 10, 14 and 17. The CBCL/4–18 is a commonly used dimensional measure of child behavior during the previous six months. The complete questionnaire contains 118 items and shows good internal reliability and validity in several population settings (Achenbach, 1991). The attention problem subscale measures both problems of attention, impulsivity and hyperactivity and consists of 11 items rated on a 3‐level Likert scale. CBCL‐AP also has moderate validity as a screening tool for combined type ADHD (inattentive and hyperactive/impulsive) (Raiker et al., 2017; Skarphedinsson et al., 2021). Teacher ratings at age 10 were available through the teacher report form (TRF) and were used to confirm the sex‐differences and clinical significance of primary findings. In a clinical context effect sizes for, a dichotomised outcome (e.g. likely diagnosis) may be more informative; therefore, TRF attention problems were transformed into T‐scores as done in the clinic, and a T‐score above 67 was considered a case (a T‐score of 65–70 is considered “borderline”). Finally, we used CBCL externalising raw scores to examine the specificity of our models. Externalising score measures delinquent and aggressive behavior and is often comorbid with attention problems (Kuja‐Halkola et al., 2015).

### Exposure variables

Offspring sex, length, abdominal circumference (AC), HC and BW were measured at birth with the latter measures considered proxies of fetal growth. For one participant sex was gathered from a later questionnaire. Gestational week of delivery was determined from last menstrual period and week 18 ultrasound. If there was more than 7 days difference the latter was chosen. We utilised the metric proportion of optimal birthweight (POBW) to estimate expected versus observed fetal growth. POBW is an estimate of fetal growth that adjusts BW for fetal sex, maternal height, maternal parity and gestational age and was developed in the Raine Study using a non small for gestational age (SGA) reference cohort. It is suggestive that POBW can reclassify constitutionally small fetuses that have met their full growth potential (Blair et al., 2005; Pereira et al., 2012).

With asymmetric fetal growth restriction, the rate of growth of the fetal AC is less than expected–this is reflected by a higher HC/AC ratio and a lower ponderal index (PI) than seen with symmetric fetal growth. Conversely, in pregnancies complicated by diabetes, there can be accelerated rates of fetal growth reflected by lower HC/AC ratios and higher *p*I. PI is a measure of asymmetric growth and was calculated using the formula $PI=100*\frac{Birthweight\left[g\right]}{{crown-to-heel\;length}^{3}\left[cm\right]}$ (smaller values indicate skinny babies, larger values indicate well‐fed babies). The ratio of HC to AC is also a measure of asymmetric growth and was obtained with simple division.

### Covariates

We included prenatal covariates that have previously been reported to have associations with maternal ADHD and fetal growth. Recent publications have highlighted an increase in prenatal risk behavior in maternal ADHD (Havdahl et al., 2022), and such behaviors may confound the relationship between fetal growth and attention problems. Prenatal questionnaire data at gestational week 16–18 provided information on maternal smoking, alcohol consumption, race, family income and education level and medical history including hypertension, diabetes and psychiatric history (e.g. “*Has any doctor ever told you that you had any of the following (0* = *No, 1* = *Yes) Psychiatric disorder _* “). Perinatal variables included gestational age at birth and APGAR score at 5 min assessed by midwifes. For maternal education we attempted to treat it as both a multilevel factor, a continuous and a dichotomized variable (more vs. less than 12 years of formal schooling), with neither approach resulting in inclusion for the final parsimonious model. The cohort CBCL‐AP assessments took place in 5 rounds, around ages five, eight, 10 14 and 17. These age measures (“age at assessment”) were treated as continuous for the interaction analysis.

### Statistics

All statistical analyses and graph production were performed in *R* (R Core Team, 2021) and its associated libraries “sjPlot”, “lme4”, “lmeresampler”, and “lmertest”. The library “ordinal” was used for sensitivity analyses of cumulative link mixed modeling (CLMM) (Christensen, 2019).

We inspected continuous variables for gross violations of normality and outliers using standard qq‐plots and histograms in both sexes. As regression using ratios can be prone to error, we used Grubbs test for HC/AC and PI to exclude any significant outliers (*n* = 2 and 3 respectively). Fetal growth parameters were normalised to ease comparison between effect sizes. Wilcoxon summed rank was used to compare sample distributions in the outcome CBCL‐AP between sexes at different ages due to non‐normal distribution.

For linear mixed modeling of repeated measures, we used the CBCL‐AP raw scores. The CBCL‐AP scores in our dataset had 20 levels (0–19 points on the CBCL‐AP) and displayed a right‐skewed non‐gaussian distribution, with most participants scoring 0. Recent work has demonstrated that the treatment of ordinal data as continuous does not impact inference in most situations (Robitzsch, 2020). Linear mixed modeling is robust to missing data, violations of distributional assumptions, heteroscedasticity of residuals, and non‐normal random‐effects (Schielzeth et al., 2020). We treated the CBCL‐AP as a continuous variable for the primary analysis to ease interpretation. Models diagnostics were evaluated by examining histograms and qq‐plots of residuals. We built our model with clustering at the participant ID level and included the neonatal anthropometric measures, as individual predictors. First we made univariable models, then included all maternal baseline variables presented in Table 1. For every neonatal anthropometric measure we used backwards variable selection for variables with a *p*‐value below 0.2 to develop our parsimonious model. Gestational age is adjusted for in POBW and was excluded from this model development. Finally we examined if a sex‐interaction improved model fit of the neonatal anthropometric measures.

**TABLE 1:** Demographics of the analytic cohort (*n* = 2387).

	Female	Male
Number of participants	1156	1231
Maternal age at birth (years)		
Mean (SD)	28.6 (±5.9)	28.5 (±5.7)
Missing	31 (2.7%)	21 (1.7%)
Income level^a^		
Mean (SD)	3.7 (±1.2)	3.7 (±1.2)
Missing	98 (8.5%)	70 (5.7%)
Maternal body mass index (kg/m^2^)		
Mean (SD)	22.4 (±4.3)	22.4 (±4.2)
Missing	30 (2.6%)	21 (1.7%)
Maternal race		
European descent	1001 (86.6%)	1092 (88.7%)
Other	125 (10.8%)	119 (9.7%)
Missing	30 (2.6%)	20 (1.6%)
Paternal race		
European descent	1001 (86.6%)	1096 (88.7%)
Other	125 (10.8%)	119 (9.6%)
Missing	30 (2.6%)	21 (1.7%)
Maternal level of education^b^		
Mean (SD)	1.2 (±1.5)	1.2 (±1.5)
Missing	30 (2.6%)	20 (1.6%)
Diabetes or hypertension in pregnancy		
Absent	938 (81.1%)	995 (80.7%)
Present	187 (16.3%)	218 (17.5%)
Missing	30 (2.6%)	20 (1.6%)
Gestational age at birth (weeks)		
Mean (SD)	38.7 (±2.4)	38.8 (±2.1)
Missing	2 (0.2%)	2 (0.2%)
Any smoking in pregnancy^c^		
Mean (SD)	0.7 (±1.3)	0.5 (±1.2)
Missing	120 (10.4%)	105 (8.2%)
Any maternal psychiatric illness		
Absent	1103 (95.4%)	1184 (96.2%)
Present	23 (2.0%)	27 (2.2%)
Missing	30 (2.6%)	20 (1.6%)
Maternal alcohol consumption in first 3 months^d^		
Mean (SD)	4.8 (±1.4)	4.8 (±1.3)
Missing	30 (2.6%)	20 (1.7%)
Apgar score at 5 min		
Mean (SD)	9.0 (±0.8)	9.0 (±0.7)
Missing	6 (0.5%)	10 (0.8%)

^a^ Family income: 1 = Less than $7000, 2 = $7000–$11,999, 3 = $12,000–$23,999, 4 = $24,000–$35,000, 5 = $36,000 or more.

^b^ Maternal Education: 0 = None or ‘other’, 1 = Trade certificate or apprenticeship, 2 = Professional registration (non‐degree), 3 = College diploma or degree, 4 = University degree.

^c^ Maternal smoking 0 = None, 1 = 1 to 5 daily, 2 = 6 to 10 daily, 3 = 11 to 15 daily, 4 = 16 to 20 daily, 5 = 21 or more per day.

^d^ Maternal alcohol consumption 1 = Daily, 2 = Several times per week, 3 = Approximately once per week, 4, Less than once per week, 5 = One binge effort, 6 = Never.

Having derived our models, we determined if the overall relationship in both sexes was linear or quadratic by comparing the Akaike Information Criterion (AIC) of models with a linear term only or linear and quadratic term. We used the parsimonious model with best fit to explore the sex stratified relationship between neonatal anthropometric measures and CBCL‐AP. We included an age‐interaction to test temporal stability. If there was no significant interaction, we concluded that the simpler model with age of assessment as an isolated fixed effect was superior.

After finding skew in the model quality control, we performed a non‐parametric bootstrap at the participant‐ID level with 2000 simulations (Thai et al., 2013). To strengthen the validity of the primary research question of an age‐interaction, we performed a sensitivity analysis with a cumulative link mixed model (CLMM) treating the CBCL‐AP as an ordinal variable. For purposes of hypothesis generation, we used a modified alpha of 0.1. The multiple steps in our analytic pathway examining several correlated exposures would strictly mandate a very conservative significance threshold. In light of the exploratory nature of our investigation we opted to maintain a liberal significance threshold based on the 95% confidence intervals.

We carried out sensitivity analysis with inference from linear mixed modeling without bootstrapping, as effect estimates are generally unaffected by assumption violations (Schielzeth et al., 2020). We used the final model from the primary analysis with exclusion of premature babies (<37 weeks) to ensure the effect was not driven by preterm neonates. To investigate if a second rater agreed with the parent rating, we analyzed teacher‐rated CBCL‐AP at age 10 using logistic regression to determine the likelihood for having clinically significant CBCL‐AP (T‐score >67). Finally, externalising raw scores were used to test the specificity of the linear mixed effects models for CBCL‐AP. Graphs were derived by applying the parsimonious model to first childhood, then adolescent behavioral assessments in males and females respectively. For the quadratic model of HC/AC we tested if terms of HC and 1/AC seperately performed similarly or changed the HC/AC‐estimates to guard against spurious correlations when using a ratio (Kronmal, 1993).

## RESULTS

A total of 2979 dyads resulted in the birth of 2868 Gen2 Raine Study participants, of which 2387 participants had at least one neonatal anthropometric measures and completed follow‐up of at least one CBCL score between ages 5–17 years (Flowchart suppl. Figure 1). Missing value percentages for the maternal baseline variables are available in Table 1. When the analysis cohort was compared to the original cohort, the analysis cohort had higher socioeconomic status, higher offspring BW and lower prevalence of prenatal risk factors such as smoking (Supplementary Table 1). When the analytic cohort was stratified by offspring sex, there was a slight increase in smoking among mothers with female offspring (Table 1), with the remaining maternal and paternal baseline variables being similar between offspring sex. Among the primary exposures of interest, BW, HC, and HC/AC were significantly lower in females than males, 145 g, 7 mm and 0.011, respectively (Table 2). PI was lower in males than females (*p*‐value = 0.06). POBW did not differ significantly between the sexes (Table 2). A total of 9385 valid CBCL‐AP assessments were available for analysis. Missing values for the behavioral assessments increased with age, and only 1111 participants had an assessment at all timepoints (564 males, and 547 females). Values differed significantly between the sexes with males showing higher CBCL‐AP across childhood and adolescence (mean from 3.6 to 2.0) compared to females (mean from 1.6 to 2.7) (Table 2 and Suppl Figure 1).

**FIGURE 1:**
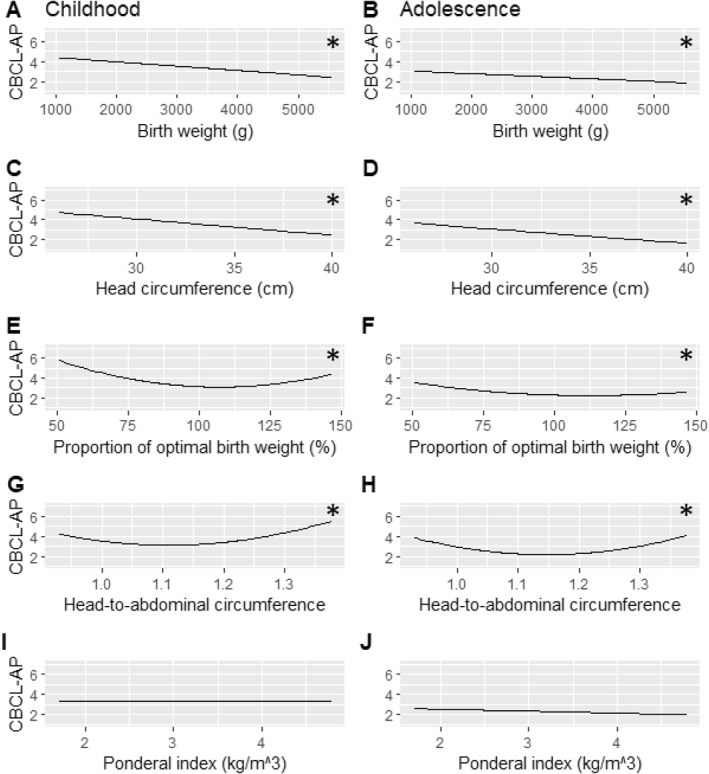
Visual aid illustrating the relationship between a given measure of neonatal anthropometry and the average parent‐rated attention problems (CBCL‐AP) in males. Childhood (assessments at ages 5, 8 and 10) is displayed on the left, and adolescence (assessments at age 14 and 17) on the right. * Highlights models with significant associations with attention problems.

**TABLE 2:** Fetal growth parameters (exposures) and CBCL attention problems (outcome) for the analytic cohort (*n* = 2387).

	Female	Male	*p*‐value
Participants	1156	1231	
Birth weight (g)			**<0.0001**
Mean (SD)	3225.4 (±605.6)	3374.8 (±598.0)	
Missing	1 (0.1%)	1 (0.1%)	
Proportion of optimal birth weight (%)			0.72
Mean (SD)	97.3 (±12.8)	97.3 (±12.7)	
Missing	8 (0.7%)	6 (0.5%)	
Head circumference (cm)			**<0.0001**
Mean (SD)	34.0 (±1.9)	34.7 (±1.8)	
Missing	20 (1.7%)	12 (1.0%)	
Ponderal index (g/cm^3^)			0.06
Mean (SD)	2.8 (±0.3)	2.8 (±0.3)	
Missing	16 (1.4%)	12 (1.0%)	
Head‐to‐abdominal circumference (ratio)			**0.00003**
Mean (SD)	1.1 (±0.1)	1.1 (±0.1)	
Missing	105 (9.1%)	92 (7.5%)	
CBCL attention problems age 5			**<0.0001**
Mean (SD)	2.7 (±2.8)	3.6 (±3.1)	
Missing	106 (9.2%)	116 (9.4%)	
CBCL attention problems age 8			**<0.0001**
Mean (SD)	2.5 (±2.9)	3.7 (±3.5)	
Missing	151 (13.1%)	163 (13.2%)	
CBCL attention problems age 10			**<0.0001**
Mean (SD)	2.0 (±2.9)	3.2 (±3.4)	
Missing	186 (16.1%)	189 (15.4%)	
CBCL attention problems age 14			**<0.0001**
Mean (SD)	1.9 (±2.6)	2.7 (±3.2)	
Missing	283 (24.5%)	321 (26.1%)	
CBCL attention problems age 17			**0.0006**
Mean (SD)	1.6 (±2.4)	2.0 (±2.7)	
Missing	489 (42.3%)	546 (44.4%)	

Bold indicates significant differences using Wilcoxon signed‐rank test. *Missing indicates the percentage of people from the age 5–17 cohort with a missing value for the given variable.

First, we examined if a second‐order polynomial improved model fit in unstratified univariable, multivariable and parsimonious models. For BW, HC and PI the AIC was lower with a linear model, whereas POBW and HC/AC demonstrated a quadratic relationship. Backwards variable removal did not result in notable changes of effect‐sizes compared to the full model (Table 3). Save for PI, male sex significantly interacted with the neonatal measures and reduced the AIC. For POBW this was related primarily to the linear term. For consistency, PI was also included in the sex‐stratified primary analysis.

**TABLE 3:** Unstratified regressions treating fetal growth as a linear or quadratic term and checking interaciton.

Neonatal anthropometric measure	Univariable regression		Full multivariable regression		Parsimonious regression		Parsimonious model interaction from male sex on neonatal anthropometry
	Linear model	Quadratic model	Linear model	Quadratic model	Linear model	Quadratic model	
BW^a^	**β: −0.13** **95%CI: [−0.25, −0.02]** **AIC: 43,481**	β: 0.00 95%CI: [−0.06, 0.06] AIC: 43,483	**β: −0.11** **95%CI: [−0.29, 0.04]** **AIC: 37,342**	β: 0.02 95%CI: [−0.07, 0.10] AIC: 37,344	**β: −0.11** **95%CI: [−0.27–0.05]** **AIC: 37,335**	β: 0.02 95%CI: [−0.07, 0.10] AIC: 37,337	**Linear β: −0.264** **95%CI: [−0.46, −0.07]** **AIC: 37,333**
HC^b^	**β: −0.16** **95%CI: [−0.28, −0.05]** **AIC: 42,908**	β: −0.01 95%CI: [−0.06, 0.04] AIC: 42,910	**β: −0.24** **95%CI: [−0.40, −0.08]** **AIC: 36,907**	β: 0.02 95%CI: [−0.06, 0.10] AIC: 36,909	**β: −0.24** **95%CI: [−0.40, −0.08]** **AIC: 36,900**	β: 0.02 95%CI: [−0.06, 0.10] AIC: 36,901	**Linear β: −0.232** **95%CI: [−0.42, −0.04]** **AIC: 36,898**
POBW^c^	β: −0.11 95%CI: [−0.22, 0.00] AIC: 43,238	**β: 0.07** **95%CI: [0.00, 0.13]** **AIC: 43,236**	β: −0.0623 95%CI: [−0.16 0.03] AIC: 37,306	**β: 0.0598** **95%CI: [−0.00, 0.12]** **AIC: 37,305**	β: −0.0704 95%CI: [−0.16, 0.02] AIC: 37,298	**β: 0.06** **95%CI: [−0.00, 0.12]** **AIC: 37,297**	**Linear β: −0.254** **95%CI: [−0.43 −0.08]** **Quadratic β: 0.0807** **95%CI: [−0.05, 0.201]** **AIC: 37,295**
HCAC^d^	β: 0.04 95%CI: [−0.07, 0.15] AIC: 39,912	**β: 0.08** **95%CI: [0.00, 0.16]** **AIC: 39,910**	β: 0.00 95%CI: [−0.12–0.11] AIC: 35,202	**β: 0.08** **95%CI: [0.00, 0.15]** **AIC: 35,197**	β: 0.01 95%CI: [−0.12, 0.11] AIC: 35,223	**β: 0.08** **95%CI: [0.00, 0.16]** **AIC: 35,219**	**Linear β: 0.0754** **95%CI: [−0.11, 0.26]** **Quadratic β: 0.157** **95%CI: [0.02, 0.30]** **AIC: 35,218**
PI^e^	**β: −0.15** **95%CI: [−0.26, −0.03]** **AIC: 42,951**	β: 0.01 95%CI: [−0.06, 0.09] AIC: 42,953	**β: −0.04** **95%CI: [−0.17, 0.08]** **AIC: 36,898**	β: 0.01 95%CI: [−0.07, 0.09] AIC: 336,900	**β: −0.06** **95%CI: [−0.18, 0.07]** **AIC: 36,960**	β: 0.01 95%CI: [−0.07, 0.10] AIC: 36,962	β: 0.00333 95%CI: [−0.17, 0.17] AIC: 37,011

*Note*: Model selection of quadratic versus linear relationship between fetal growth parameters and CBCL attention problems. For quadratic models, only the effect estimate and confidence interval for the quadratic term is shown. Bold indicates the model chosen based on AIC. Full multivariable model adjusted for fetal sex, age at CBCL assessment, gestational age at birth, maternal pregnancy variables (age, race/ethnicity, smoking, education, BMI, psychiatric history, alcohol consumption, diabetes or hypertension) and family income. For POBW fetal sex and gestational age at birth were excluded.

Abbreviations: AIC, Akaike Information Criterion; BW, birth weight; HC, head circumference; HCAC, head‐to‐abdominal circumference; PI, Ponderal index; POBW, proportion of optimal birth weight.

^a^ Parsimonious *n* = 2053 and model adjusted for age at assessment, maternal age, gestational age at birth, smoking in pregnancy, income level, maternal BMI, maternal race and APGAR score at 5 min.

^b^ Parsimonious *n* = 2029 and model adjusted for age at assessment, maternal age, gestational age at birth, smoking in pregnancy, income level, maternal BMI and APGAR score at 5 min.

^c^ Parsimonious *n* = 2051 and model adjusted for age at assessment, maternal age, smoking in pregnancy, income level, maternal BMI and APGAR score at 5 min.

^d^ Parsimonious *n* = 1943 and model adjusted for age at assessment, maternal age, gestational age at birth, smoking in pregnancy, income level, maternal BMI.

^e^ Parsimonious *n* = 2029 and model adjusted for age at assessment, maternal age, gestational age at birth, smoking in pregnancy, income level, maternal BMI.

The original primary model‐output can be seen in supplementary Table 2 but confidence intervals were derived with bootstrapping as inspection of residuals showed violation of distributional assumptions (Table 4 and Table 5).

**TABLE 4:** Final parsimonious models for the effects of fetal growth on ADHD in males.

Fetal growth parameter	Multivariable model with age^a^ Fetal growth parameter‐interaction	Multivariable model with age^a^ Fetal growth parameter‐interaction	Multivariable model without age‐interaction
	Age‐interaction shown	Fetal growth parameter shown	Fetal growth parameter shown
BW (*n* = 1082)^a^	β: 0.0184 95%CI: [−0.006, 0.0414]	β: −0.425 95%CI: [−0.731, −0.104]	**β: −0.234** **95%CI: [−0.422, −0.0497]**
HC (*n* = 1073)^a^	β: 0.0111 95%CI: [−0.0153, 0.0363]	β: −0.432 95%CI: [−0.744, −0.107]	**β: −0.316** **95%CI: [−0.495, −0.133]**
POBW (*n* = 1082)^b^	1. Order β: 0.0161 95%CI: [−0.00646, 0.0364] 2. Order β: −0.00800 95%CI: [−0.0229, 0.00760]	1. Order β: −0.343 95%CI: [−0.607, −0.0569] 2. Order β: 0.191 95%CI: [−0.00788, 0.380]	**1. Order** **β: −0.175** **95%CI: [−0.317, −0.0339]** **2. Order** **β: 0.106** **95%CI: [0.0194, 0.191]**
HCAC (*n* = 1027)^c^	1. Order β: −0.0185 95%CI: [−0.0400, 0.00168] 2. Order β: −0.00792 95%CI: [−0.0196, 0.00665]	1. Order β: 0.187 95%CI: [−0.0766, 0.464] 2. Order β: 0.208 95%CI: [−0.00286, 0.384]	**1. Order** **β: −0.00324** **95%CI: [−0.154, 0.146]** **2. Order** **β: 0.126** **95%CI: [0.0145, 0.238]**
PI (*n* = 1073)^c^	β: −0.0200 95%CI: [−0.298, 0.246]	β: 0.0140 95%CI: [−0.249, 0.282]	β: −0.0487 95%CI: [−0.184, 0.0855]

*Note*: Age interaction and effect estimates for the association between fetal growth parameters and attention problems in males. Bold indicates significant results at the 0.05 level.

Abbreviations: BW, birth weight; HC, head circumference; HCAC, head‐to‐abdominal circumference; PI, Ponderal index; POBW, proportion of optimal birth weight.

^a^ Adjusted for age at assessment, maternal age, gestational age at birth, smoking in pregnancy, income level, maternal BMI, maternal race and APGAR score at 5 min.

^b^ Adjusted for age at assessment, maternal age, smoking in pregnancy, income level, maternal BMI and APGAR score at 5 min.

^c^ Adjusted for age at assessment, maternal age, gestational age at birth, smoking in pregnancy, income level, maternal BMI.

**TABLE 5:** Final parsimonious models for the effects of fetal growth on ADHD in females.

Fetal growth parameter	Multivariable model with age^a^ Fetal growth parameter‐interaction	Multivariable model with age^a^ fetal growth parameter‐interaction	Multivariable model without age‐interaction
	Age‐interaction shown	Fetal growth parameter shown	Fetal growth parameter shown
BW (*n* = 971)^a^	β: 0.0150 95%CI: [−0.005, 0.04]	β: −0.116 95%CI: [−0.405, 0.166]	β: 0.0430 95%CI: [−0.129, 0.225]
HC (*n* = 956)^a^	**β: 0.0272** **95%CI: [0.006, 0.05]**	**β** **: −0.424** **95%CI:** **[** **−0.726, −0.133** **]**	β: −0.136 95%CI: [−0.303, 0.0377]
POBW (*n* = 969)^b^	1. Order β: −0.00169 95%CI: [−0.0187, 0.0167] 2. Order β: −0.001 95%CI: [−0.0146, 0.0124]	1. Order β: 0.0619 95%CI: [−0.185, 0.294] 2. Order β: 0.0232 95%CI: [−0.148, 0.209]	1. Order β: 0.0441 95%CI: [−0.0862, 0.177] 2. Order β: 0.0137 95%CI: [−0.0805, 0.117]
HCAC (*n* = 916)^c^	1. Order β: 0.0114 95%CI: [−0.00604, 0.0274] 2. Order β: 0.000675 95%CI: [−0.0115, 0.0105]	1. Order β: −0.180 95%CI: [−0.401, 0.0532] 2. Order β: 0.0179 95%CI: [−0.121, 0.179]	1. Order β: −0.0606 95%CI: [−0.190, 0.0614] 2. Order β: 0.0250 95%CI: [−0.0657, 0.114]
PI (*n* = 960)^c^	β: −0.0156 95%CI: [−0.262, 0.235]	β: −0.00510 95%CI: [−0.0248, 0.0150]	β: −0.0693 95%CI: [−0.185, 0.0549]

*Note*: Age interaction and effect estimates for the association between fetal growth parameters and attention problems in females. Bold indicates significant results at the 0.05 level.

Abbreviations: BW, birth weight; HC, head circumference; HCAC, head‐to‐abdominal circumference; PI, Ponderal index; POBW, proportion of optimal birth weight.

^a^ Adjusted for age at assessment, maternal age, gestational age at birth, smoking in pregnancy, income level, maternal BMI, maternal race and APGAR score at 5 min.

^b^ Adjusted for age at assessment, maternal age, smoking in pregnancy, income level, maternal BMI and APGAR score at 5 min.

^c^ Adjusted for age at assessment, maternal age, gestational age at birth, smoking in pregnancy, income level, maternal BMI.

In males, there was an inverse relationship between BW and CBCL‐AP. For each 1‐SD (606.2 g) lower BW, there was a 0.234 (95%CI [−0.422, −0.0497]) higher CBCL‐AP with no age interaction. No significant relationship was seen between BW and CBCL‐AP in the females, although the age interaction trended toward significance (β: 0.0150 (95%CI [−0.005, 0.04]). Excluding the age interaction in females did not influence the relationship between BW and CBCL‐AP.

**FIGURE 2:**
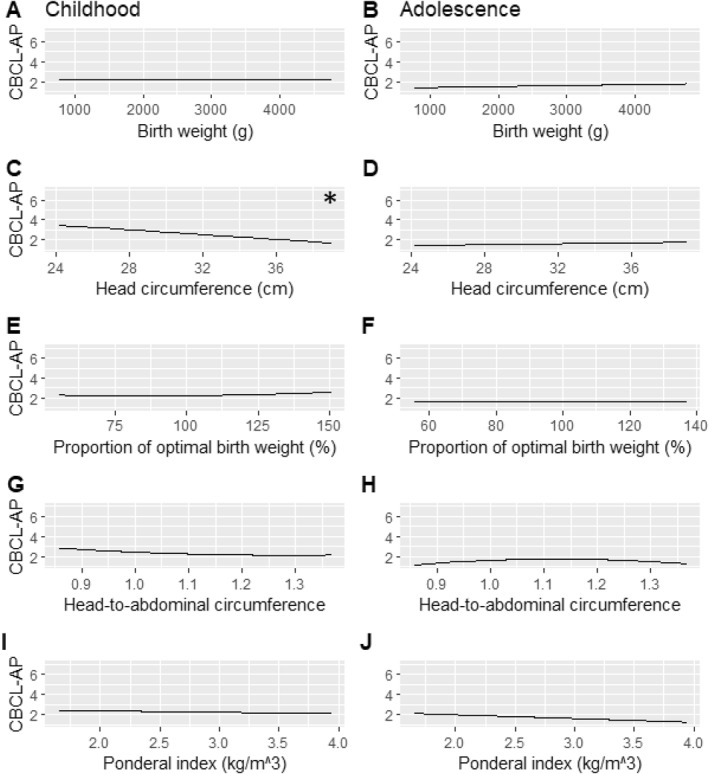
Visual aid illustrating the relationship between a given measure of neonatal anthropometry and the average parent‐rated attention problems (CBCL‐AP) in females. Childhood (assessments at ages 5, 8 and 10) is displayed on the left, and adolescence (assessments at age 14 and 17) on the right. * Highlights the significant association between head circumference and attention problems that decreased with advancing age.

In males, there was an inverse linear relationship between HC and CBCL‐AP. For each 1‐SD (1.9 cm) lower HC there was a 0.316 (95%CI [0.495, 0.133]) higher CBCL‐AP with no age interaction. In females there was also an inverse linear relationship between HC and CBCL‐AP with a 1‐SD (1.9 cm) lower HC resulting in a 0.424 (95%CI [0.726, 0.133]) higher CBCL‐AP. There was a significant interaction between HC and age in females with a 0.027 (95% *CI*: 0.006–0.05) reduction in CBCL‐AP effect size per year (Table 5 and Figure 2).

In males, there was a quadratic relationship between POBW and CBCL‐AP with no age interaction. After excluding the age interaction, the quadratic relationship between POBW and CBCL‐AP remained significant (β: 0.106 [95% *CI* 0.0194, 0.191]) (Table 4 and Figure 1). No significant relationship between POBW and CBCL‐AP was seen in females, and there was no significant age interaction.

In males there was a quadratic relationship between HC/AC and CBCL‐AP with no age interaction. After excluding the age interaction, the quadratic relationship between HC/AC and CBCL‐AP remained significant (β: 0.126 [95% *CI* 0.0145, 0.238]) (Table 4 and Figure 1). No significant relationship between HC/AC and CBCL‐AP was seen in females, and there was no significant age interaction (Table 5). In males and females there was no association between PI and CBCL‐AP (Table 4 and Table 5).

We performed a sensitivity analysis treating the CBCL‐AP as an ordinal variable in a CLMM. This approach did not meaningfully change the age interaction for BW, POBW, HC, or HC/AC. The age‐interacton for the HC model in females was only borderline significant with this approach, but the directionality and effect size was consistent with primary results (Supplementary Table 3).

The rate of preterm birth (gestational week <37) was relatively low in this study (*N* = 258 of the 2387 participants) and sensitivity analysis excluding preterm birth did not alter the magnitude of effect sizes for BW, HC and HC/AC (Supplementary Table 4); however, the quadratic association to POBW was no longer present.

We sought to confirm our results using a second assessor. Sensitivity analysis using a multivariable logistic model on teacher reporting of attention problems with a T‐score >67 generally agreed with the directionality and significance of primary findings; however, POBW did not seem to display a second‐order relationship with CBCL‐AP (Supplementary Table 5). Finally, we switched the outcome in the model to externalising raw scores, in order to test if the relationships were specific CBCL‐AP. Overall, the only neonatal anthropometric measures that remained significant was HC (Supplementary Table 6). *Post hoc* we investigated and found that for HC/AC, the ratio was the better predictor of CBCL‐AP and adding fixed effects of HC and 1/AC did not have a meaningful impact on effect‐estimates (Supplementary Table 7).

## DISCUSSION

Using longitudinal data across childhood and adolescence, we have demonstrated inverse and quadratic relationships between neonatal anthropometry and attention problems. Effects were larger in males than females. The relationships between neonatal anthropometry and attention problems persisted across childhood and adolescence in males.

Most larger published studies to date are consistent with male vulnerability (Dooley et al., 2022; Momany et al., 2017). Only one study found that females were at increased risk (Murray et al., 2015). We did find an effect‐reduction with increasing age in females, and the age‐four assessment of the PELOTAS‐cohort could reflect stronger initial effects in females; however, assessment of CBCL‐AP in our cohort at age 5 was consistent with an increased male affliction. The differences are likely not caused by statistical methodology, as an ordinal approach did not meaningfully change inference. Cultural differences between Brazil and Australia may influence parent reporting of symptoms because of different perceptions of gender‐specific “normal behavior”, as seen in other ethnic groups (Javo et al., 2000). Lastly, differences in maternal baseline characteristics relating to education, income, preexisting mental illness, ethnicity and BMI could influence the associations seen between neonatal anthropometric and CBCL‐AP in the two cohorts.

There are several potential mechanisms for the bigger effect sizes of neonatal anthropometry in males as it relates to fetal growth. A recent analysis from the SCOPE‐study found that SGA males had more asymmetric growth than SGA females (van der Vlugt et al., 2020) possibly suggesting that males are more prone to developmental programming. Longitudinal MRI‐studies of fetal brain development have highlighted a larger male growth rate of white matter in fronto‐temporal and parite‐occipital regions during weeks 18–36 of gestation (Studholme et al., 2020), corresponding to white matter abnormalities seen in ADHD (Silk et al., 2009). As most fetal growth also occurs during this time, male fetuses could be particularly vulnerable to neurodevelopmental adversity compared to females. In addition it has been suggested that genetic propensity toward ADHD enhances the effect of fetal growth on ADHD symptoms (Rahman et al., 2021). This could also apply to the higher propensity for attention problems in individuals with a y‐chromosome. Alternatively, maternal stress and cortisol has been associated with birth weight and could underlie the associations reported in our study (Bussières et al., 2015; Cherak et al., 2018). Animal studies in guinea pigs have suggested a male vulnerability to hyperactive behavior from prenatal stress and cortisol dysregulation through reduced myelination and disruption of GABAergic signaling (Crombie et al., 2021).

Others have argued, that a shorter gestational duration increases the risk of ADHD diagnosis (Robinson et al., 2022) and so could confound the association with neonatal anthropometry; however, the Norwegian Mothers and Babies Cohort (MOBA) found that such effects were isolated to females (Ask et al., 2018). Together with our findings, this also raises the possibility that comorbidity of poor fetal growth and preterm birth mask sex differences arising from one or the other. We further attempted to address this by including gestational age in our models, analysing POBW and excluding preterm birth in sensitivity analysis. The relationship between neonatal anthropometry appeared robust suggesting effects of fetal growth persisted in males compared to females. Potential explanations for the age‐dependent manifestation in females include changes brought on by puberty or simply a small effect size not detected in our dataset. From a teleological perspective, the potential adaptive advantage of an impulsive and hyperactive phenotype may be reduced in adolescent females because of the parental demands of childrearing; however, replication of this age‐dependent decline in effect is needed before putting it in an evolutionary context.

In this study the inverse linear effect of HC was 50% bigger than the effect of BW. Previous reports support a larger effect size from reductions of HC, and one study found that the effect of HC remained after adjustment for BW (Aagaard et al., 2018; Murray et al., 2015). HC correlates with brain volume in neonates (Lindley et al., 1999), and brain volume is associated with ADHD with regional differences explaining symptom severity (Castellanos et al., 2002). In contrast, the relationship between measures of HC/AC and POBW appears to be quadratic. To our knowledge, the only other study that found a quadratic relationship between fetal growth and attention problems is Van Mil et al., which found that high offspring BW was associated with increased risk of CBCL‐AP in obese mothers. Our results appear to replicate this finding, as POBW is adjusted for maternal characteristics and our model included maternal BMI. Importantly, the effect of POBW was not present in two sensitivity analyses. In contrast the quadratic relationship between CBCL‐AP and HC/AC was robust and u‐shaped. The potential mechanisms between reduced HC/AC and CBCL‐AP is unclear and needs replication; however, there was no effect of PI hinting that not all asymmetry predicts risk. Instead, increased relative organogenesis of the abdomen compared to the head is associated with neurodevelopmental adversity.

The effect sizes of neonatal anthropometry on CBCL‐AP for the individual is challenging to interpret. We get a large effect if we divide the parameter estimates by the sample mean. A 1‐SD decrease in HC for females at age 5 corresponds to approximately a 10% increase in average attention problems. Considering instead a scale from 0 to 19, the effect sizes are modest (all <1 per SD). When examining the teacher‐sensitivity analysis using T‐score> 67 the effect size was considerable (OR = 0.59 per SD higher HC), but the lack of repeated measures, varants caution as this estimate has a larger degree of uncertainty. Finally, evidence from Rahman et al. has suggested that effects of LBW on ADHD symptoms vary by genetic risk (Rahman et al., 2021). Our sample size was not strong enough to do a varying slope varying intercepts regression, which might have offered some insight into individual differences in the effects of fetal growth. Our supplementary analysis of externalising problems suggest that the final modeling of CBCL‐AP is not necessarily generalisable to other types of behavior typically comorbid with attention problems, although the model of HC remained significant. As our model and covariable selection related to CBCL‐AP this should not be interpreted as a specific examination of externalising behavior, but was meant as an examination of our models specificity for CBCL‐AP.

Our study has several strengths. It is relatively large, longitudinal and has good retention (84%). We had a single assessor (parents) for all our measures in the primary analysis and could confirm results with a second assessor (teachers). CBCL is well‐validated and available in many different countries. Our study also has important limitations. Despite a relatively large sample size, we had findings that bordered on significance, and increased power may have altered the primary results and our overall interpretation. In addition, the many fetal growth parameters used to inform the relationship between fetal growth and attention problems run the risk of type 1 error as we did not correct for multiple comparison. Finally, our analytic cohort was subject to selective attrition of the least affluent participants which could potentially bias results, and the generalisability was limited by the primarily Caucasian cohort and selection from a westernised society.

Future research should examine the neurobiology that underlie increases in attention problems from fetal growth; furthermore, exploration of prenatal growth as assessed with ultrasound could uncover gestational periods critical to development of higher CBCL‐AP, whereas studies of growth in the first years following birth could identify potential windows of opportunity to help mitigate effects of fetal growth. Finally, the sex‐specific effects of fetal growth should be confirmed with genetic epidemiology.

## AUTHOR CONTRIBUTIONS

**Lars Meinertz Byg**: Conceptualization; Formal analysis; Visualization; Writing – original draft; Writing – review & editing. **Carol Wang**: Formal analysis; Methodology; Writing – review & editing. **Jonathan Hirst**: Writing – review & editing. **Roger Smith**: Writing – review & editing. **Craig Pennell**: Conceptualization; Writing – review & editing.

## CONFLICT OF INTEREST STATEMENT

The authors declare no conflicts of interest.

## ETHICAL CONSIDERATIONS

Ethics approval was not required as data were publicly available and de‐identified.

## ACCESS TO DATA AND DATA SHARING

The first and second authors of this paper had full access to all the data in the study and take responsibility for the integrity of the data and the accuracy of the data analysis.

## Supporting information

Supporting Information S1

## Data Availability

Our data is available only to researchers with approval from the Raine study steering group.
