# Supplementary material for: The longitudinal effects of neonatal anthropometry on attention problems in males and females
Source: JCPP Adv. 2024 Jul 29;5(2):e12256. doi: 10.1002/jcv2.12256 (PMC12159305; doi:10.1002/jcv2.12256)
Supplement: Supplementary file 1 — Supporting Information S1 [file JCV2-5-e12256-s001.docx]

**Supplementary figure 1** Flowchart of cohort selection


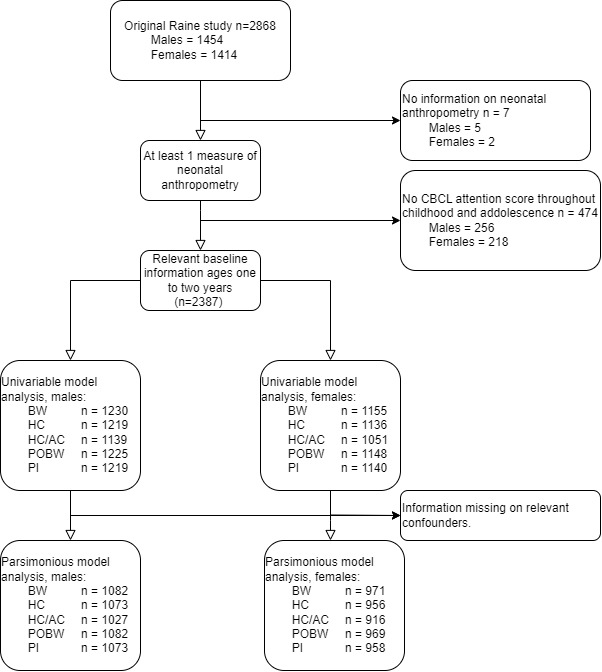


Supplementary figure 2. The cohort selection detailing excluded participants, and participant numbers used in the univariable, and parsimonious models. Different sets of covariables were used for parsimonious models and the excluded participants therefore varied at the final step.

**Supplementary figure 2**. Distribution of CBCL attention problems scores in males and females

Supplementary figure 2. Violin plots showing score distribution across the five assessments. The black line indicates the mean score and shows the gradual decrease with increasing age


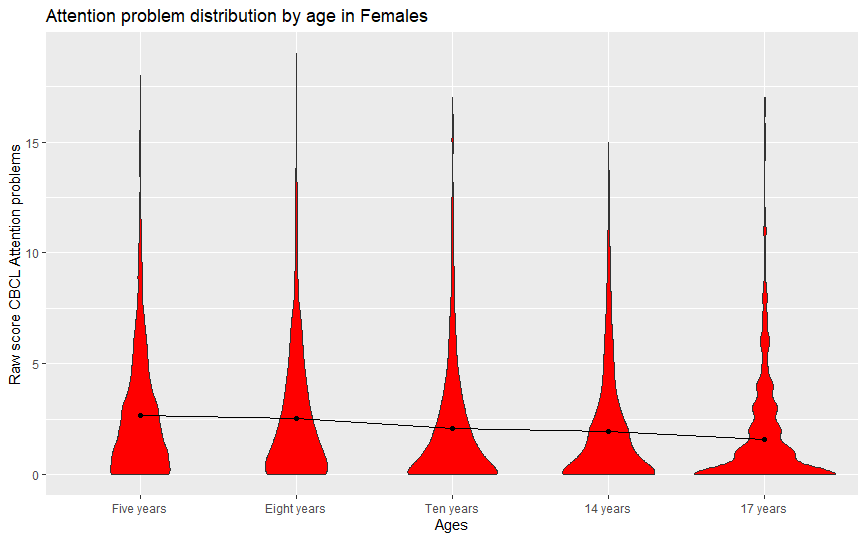

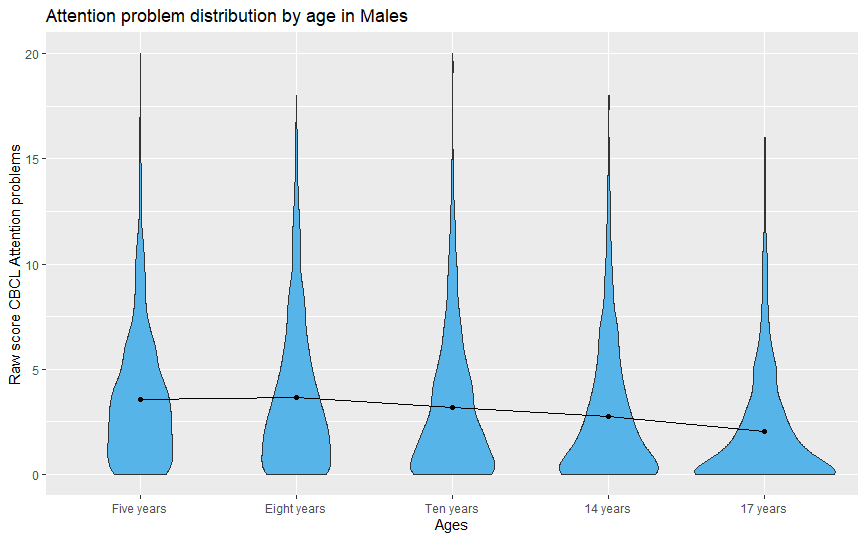


| Supplementary table 1: Demographics for the original Raine Study cohort (n = 2868) | | |
| --- | --- | --- |
|  | **Female** | **Male** |
| **Number of participants** | 1414 | 1454 |
| **Maternal age at birth (years)** |  |  |
| Mean (SD) | 28.0 (±6.0) | 28.1 (±5.9) |
| Missing | 39 (2.8%) | 33 (2.3%) |
| **Income level†** |  |  |
| Mean (SD) | 3.6 (±1.2) | 3.6 (±1.3) |
| Missing | 133 (9.4%) | 97 (6.7%) |
| **Maternal body mass index (kg/m^2)** |  |  |
| Mean (SD) | 22.4 (±4.4) | 22.3 (±4.3) |
| Missing | 36 (2.5%) | 29 (2.0%) |
| **Maternal race** |  |  |
| European descent | 1,199 (84.8%) | 1,273 (87.6%) |
| Other | 179 (12.7%) | 153 (10.5%) |
| Missing | 36 (2.5%) | 28 (1.9%) |
| **Maternal level of education††** |  |  |
| Mean (SD) | 1.2 (±1.5) | 1.1 (±1.5) |
| Missing | 36 (2.5%) | 28 (1.9%) |
| **Diabetes or hypertension in pregnancy** |  |  |
| absent | 1,148 (81.2%) | 1,183 (81.4%) |
| present | 230 (16.3%) | 243 (16.7%) |
| Missing | 36 (2.5%) | 28 (1.9%) |
| **Gestational age at birth (weeks)** |  |  |
| Mean (SD) | 38.6 (±2.5) | 38.7 (±2.3) |
| Missing | 4 (0.3%) | 7 (0.5%) |
| **Smoking in pregnancy†††** |  |  |
| Mean (SD) | 0.7 (±1.4) | 0.6 (±1.3) |
| Missing | 173 (12.2%) | 146 (10.0%) |
| **Any maternal psychiatric illness** |  |  |
| Absent | 1,349 (95.4%) | 1,389 (95.5%) |
| Present | 29 (2.1%) | 37 (2.5%) |
| Missing | 36 (2.5%) | 28 (1.9%) |
| **Maternal alcohol consumption in first three months††††** |  |  |
| Mean (SD) | 4.9 (±1.3) | 4.8 (±1.3) |
| Missing | 36 (2.5%) | 28 (1.9%) |
| **Maternal age at birth (years)** |  |  |
| Mean (SD) | 28.0 (±6.0) | 28.1 (±5.9) |
| Missing | 39 (2.8%) | 33 (2.3%) |
| **Income level†** |  |  |
| Mean (SD) | 3.6 (±1.2) | 3.6 (±1.3) |
| Missing | 133 (9.4%) | 97 (6.7%) |
| †Family income: 1=Less than $7,000, 2=$7,000-$11,999, 3=$12,000-$23,999, 4=$24,000-$35,000, 5=$36,000 or more  ††Education: 0 = None or `other`, 1 = Trade certificate or apprenticeship, 2=Professional registration (non-degree), 3=College diploma or degree, 4=University degree  ††† Maternal smoking: 0=None, 1=1 to 5 daily, 2=6 to 10 daily, 3=11 to 15 daily, 4=16 to 20 daily, 5=21 or more per day  ††††Maternal alcohol consumption 1=Daily, 2=Several times per week, 3=Approximately once per week, 4, Less than once per week, 5=One binge effort, 6=Never | | |

##

Supplementary table 2: The original linear mixed modelling.

| Fetal growth parameter | Male parsimonious model. Age Interaction | Male parsimonious model. Fetal growth parameter | Female parsimonious model. Age interaction | Female parsimonious model. Fetal growth parameter |
| --- | --- | --- | --- | --- |
| BW | n = 1082  OR: 0.02  95%CI: [0.00, 0.04]  P-value: 0.045 | n = 1082  OR: -0.43  95%CI: [-0.72, -0.13]  P-value: 0.005 | n = 971  OR: 0.02  95%CI: [-0.00, 0.03]  P-value: 0.073 | n = 971  OR: -0.12  95%CI: [-0.40, 0.16]  P-value: 0.418 |
| HC | n = 1073  OR: 0.01  95%CI: [-0.01, 0.03]  P-value: 0.255 | n = 1073  OR: -0.43  95%CI: [-0.74, -0.12]  P-value: 0.006 | n = 956  OR: 0.03  95%CI: [0.01, 0.05]  P-value: 0.003 | n = 956  OR: -0.42  95%CI: [-0.71, -0.13]  P-value: 0.004 |
| POBW | n = 1082  *1. Order*  OR: 0.02  95%CI: [-0.00, 0.03]  P-value: 0.054  *2. Order*  OR: -0.01  95%CI: [-0.02, 0.00]  P-value: 0.123 | n = 1082  *1. Order*  OR: -0.34  095%CI: [-0.59, -0.10]  P-value: 0.006  *2. Order*  OR: 0.19  95%CI: [0.04, 0.34]  P-value: 0.013 | n = 969  *1. Order*  OR: -0.00  95%CI: [-0.02, 0.01]  P-value: 0.828  *2. Order*  OR: -0.00  95%CI: [-0.01, 0.01]  P-value: 0.860 | n = 969   1. *Order*   OR: 0.06  95%CI: [-0.16, 0.29]  P-value: 0.589  *2. Order*  OR: 0.02  95%CI: [-0.12, 0.17]  P-value: 0.751 |
| HCAC | n = 1027  *1. Order*  OR: -0.02  95%CI: [-0.04, -0.00]  P-value: 0.033  *2. Order*  OR: -0.01  95%CI: [-0.02, 0.00]  P-value: 0.075 | n = 1027  *1. Order*  OR: 0.20  95%CI: [-0.06, 0.45]  P-value: 0.126  *2. Order*  OR: 0.28  95%CI: [0.11, 0.46]  P-value: 0.001 | n = 916  *1. Order*  OR: 0.01  95%CI: [-0.00, 0.03]  P-value: 0.131  *2. Order*  OR: 0.00  95%CI: [-0.01, 0.01]  P-value: 0.897 | n = 916  *1. Order*  OR: -0.18  95%CI: [-0.39, 0.03]  P-value: 0.098  *2. Order*  OR: 0.02  95%CI: [-0.13, 0.16]  P-value: 0.811 |
| PI | n = 1073  OR: -0.02  95%CI: [-0.28 – 0.25]  P-value: 0.882 | n = 1073  OR: -0.00  95%CI: [-0.02 – 0.02]  P-value: 0.763 | n = 958  OR: -0.01  95%CI: [-0.02 – 0.01]  P-value: 0.499 | n = 958  OR: -0.02  95%CI: [-0.23 – 0.20]  P-value: 0.889 |

Supplementary table 2 showing model output prior to bootstrap.

* Adjusted for age at assessment, maternal age, gestational age at birth, smoking in pregnancy, income level, maternal BMI, maternal race and APGAR score at 5 minutes

** Adjusted for age at assessment, maternal age, gestational age at birth, smoking in pregnancy, income level, maternal BMI and APGAR score at 5 minutes

*** Adjusted for age at assessment, maternal age, smoking in pregnancy, income level, maternal BMI and APGAR score at 5 minutes

**** Adjusted for Age at assessment, maternal age, gestational age at birth, smoking in pregnancy, income level, maternal BMI

BW = Birth weight, HC = Head Circumference, POBW = Proportion of optimal birth weight, HCAC = Head-to-abdominal circumference, PI = Ponderal index.

Supplementary table 3: Treating the CBCL-AP as an ordinary variable with a cumulative link mixed model

| Fetal growth parameter | Male parsimonious model. Age Interaction | Male parsimonious model. Fetal growth parameter | Female parsimonious model. Age interaction | Female parsimonious model. Fetal growth parameter |
| --- | --- | --- | --- | --- |
| BW | n = 1082  OR: 1.01  95%CI: [0.99 – 1.02]  P-value: 0.504 | n = 1082  OR: 0.86  95%CI: [0.72 – 1.03]  P-value: 0.098 | n = 971  OR: 1.01  95%CI: [0.99 – 1.03]  P-value: 0.177 | n = 971  OR: 0.91  95%CI: [0.74 – 1.11]  P-value: 0.344 |
| HC | n = 1073  OR: 1.00  95%CI: [0.99 – 1.02]  P-value: 0.643 | n = 1073  OR: 0.85  95%CI: [0.70 – 1.03]  P-value: 0.092 | n = 956  OR: 1.02  95%CI: [1.00 – 1.04]  P-value: 0.067 | n = 956  OR: 0.74  95%CI: [0.59 – 0.91]  P-value: 0.005 |
| POBW | n = 1082  *1. Order*  OR: 1.00  95%CI: [0.99 – 1.02]  P-value: 0.565  *2. Order*  OR: 1.00  95%CI: [0.99 – 1.01]  P-value: 0.738 | n = 1082   1. *Order*   OR: 0.89  95%CI: [0.76 – 1.04]  P-value: 0.141  *2. Order*  OR: 1.06  95%CI: [0.96 – 1.17]  P-value: 0.255 | n = 969  *1. Order*  OR: 1.00  95%CI: [0.99 – 1.02]  P-value: 0.93  *2. Order*  OR: 1.00  95%CI: [0.99 – 1.01]  P-value: 0.553 | n = 969  *1. Order*  OR: 1.03  95%CI: [0.86 – 1.23]  P-value: 0.753  *2. Order*  OR: 1.04  95%CI: [0.93 – 1.17]  P-value: 0.513 |
| HCAC | n = 1027  *1. Order*  OR: 0.99  95%CI: [0.97 – 1.00]  P-value: 0.11  *2. Order*  OR: 1.00  95%CI: [0.99 – 1.01]  P-value: 0.776 | n = 1027  *1. Order*  OR: 1.10  95%CI: [0.93 – 1.30]  P-value: 0.274  *2. Order*  OR: 1.10  95%CI: [0.98 – 1.23]  P-value: 0.111 | n = 916  *1. Order*  OR: 1.00  95%CI: [0.99 – 1.02]  P-value: 0.774  *2. Order*  OR: 1.00  95%CI: [0.99 – 1.01]  P-value: 0.709 | n = 916  *1. Order*  OR: 0.94  95%CI: [0.79 – 1.11]  P-value: 0.452  *2. Order*  OR: 1.03  95%CI: [0.91 – 1.15]  P-value: 0.73 |
| PI | n = 1073  OR: 1.00  95%CI: [0.99 – 1.02]  P-value: 0.932 | n = 1073  OR:  95%CI: []  P-value: | n = 958  OR: 0.99  95%CI: [0.98 – 1.01]  P-value: 0.498 | n = 958  OR: 1.03  95%CI: [0.86 – 1.22]  P-value: 0.757 |

Supplementary table 3 showing model output with an ordinal approach

* Adjusted for age at assessment, maternal age, gestational age at birth, smoking in pregnancy, income level, maternal BMI, maternal race and APGAR score at 5 minutes

** Adjusted for age at assessment, maternal age, gestational age at birth, smoking in pregnancy, income level, maternal BMI and APGAR score at 5 minutes

*** Adjusted for age at assessment, maternal age, smoking in pregnancy, income level, maternal BMI and APGAR score at 5 minutes

**** Adjusted for Age at assessment, maternal age, gestational age at birth, smoking in pregnancy, income level, maternal BMI

BW = Birth weight, HC = Head Circumference, POBW = Proportion of optimal birth weight, HCAC = Head-to-abdominal circumference, PI = Ponderal index.

*Final model adjusted for age at CBCL assessment, gestational age at birth, maternal age at birth, maternal smoking, income below 12 000 AUD, maternal BMI and presence of maternal psychiatric diagnosis

**Final model adjusted for age at assessment, maternal age at birth, smoking status, income below 12 000 AUD, maternal BMI, maternal psychiatric illness and APGAR-score at 5 minutes.

**Bold** indicates significant results

Supplementary table 4: Sensitivity analysis excluding preterm infants (<37 weeks gestational age at birth)

| Fetal growth parameter | Male parsimonious model. Fetal growth parameter | Female parsimonious model. Age interaction | Female parsimonious model. Fetal growth parameter |
| --- | --- | --- | --- |
| BW | N = 1007  β: -0.24  95%CI: [-0.46, 0.00] | NA | N = 894  β: -0.04  95%CI: [-0.26, 0.19] |
| HC | N = 998  β: -0.31  95%CI: [-0.56, -0.07] | N = 881  β: 0.02  95%CI: [-0.00, 0.04] | N = 881  β: -0.36  95%CI: [-0.69, -0.04] |
| POBW | N = 1006  *1. Order*  β: -0.16  95%CI: [-0.35, 0.02]  *2. Order*  β: 0.05  95%CI: [-0.07, 0.17] | NA | N = 891  *1. Order*  β: -0.03  95%CI: [-0.20, 0.14]  *2. Order*  β: -0.03  95%CI: [-0.15, 0.08] |
| HCAC | N = 959  *1. Order*  β: 0.03  95%CI: [-0.16, 0.21]  *2. Order*  β: 0.16  95%CI: [0.03, 0.29] | NA | N = 848  *1. Order*  β: -0.02  95%CI: [-0.18, 0.13]  *2. Order*  β: -0.01  95%CI: [-0.12, 0.10] |
| PI | N = 998  β: -0.06  95%CI: [-0.26, 0.13] |  | N = 885  β: -0.06  95%CI: [-0.22, 0.10] |

Supplementary table 4 with confirmation of primary analysis in the term-only cohort.

* Adjusted for age at assessment, maternal age, gestational age at birth, smoking in pregnancy, income level, maternal BMI, maternal race and APGAR score at 5 minutes

** Adjusted for age at assessment, maternal age, gestational age at birth, smoking in pregnancy, income level, maternal BMI and APGAR score at 5 minutes

*** Adjusted for age at assessment, maternal age, smoking in pregnancy, income level, maternal BMI and APGAR score at 5 minutes

**** Adjusted for Age at assessment, maternal age, gestational age at birth, smoking in pregnancy, income level, maternal BMI

BW = Birth weight, HC = Head Circumference, POBW = Proportion of optimal birth weight, HCAC = Head-to-abdominal circumference, PI = Ponderal index.

Supplementary table 5 Sensitivity analysis of teacher assessments.

| Fetal growth parameter | Male | Female |
| --- | --- | --- |
| BW | N = 869  OR: 0.57  95%CI: [0.32 – 0.99]  P-value: 0.049 | N = 765  OR: 0.69  95%CI: [0.28 – 1.66]  P-value: 0.407 |
| HC | N = 862  OR: 0.59  95%CI: [0.35 – 1.00]  P-value: 0.044 | N = 754  OR: 0.89  95%CI: [0.37 – 2.22]  P-value: 0.802 |
| POBW | N = 869  *1. Order*  OR: 0.66  95%CI: [0.40 – 1.01]  P-value: 0.074  *2. Order*  OR: 0.96  95%CI: [0.67 – 1.24]  P-value: 0.800 | N = 763  *1. Order*  OR: 0.57  95%CI: [0.20 – 1.27]  P-value: 0.209  *2. Order*  OR: 0.66  95%CI: [0.25 – 1.16]  P-value: 0.288 |
| HCAC | N = 829  *1. Order*  OR: 0.81  95%CI: [0.59 – 1.14]  P-value: 0.217  *2. Order*  OR: 1.21  95%CI: [1.01 – 1.42]  P-value: 0.023 | N = 723  *1. Order*  OR: 2.64  95%CI: [1.22 – 7.36]  P-value: 0.033  *2. Order*  OR: 0.79  95%CI: [0.48 – 1.15]  P-value: 0.282 |
| PI | N = 860  OR: 0.82  95%CI: [0.52 – 1.25]  P-value: 0.364 | N = 756  OR: 0.90  95%CI: [0.49 – 1.65]  P-value: 0.728 |

Supplementary table 5: Logistic regression of TRF T-scores > 67 using the parsimonious models

* Adjusted for age at assessment, maternal age, gestational age at birth, smoking in pregnancy, income level, maternal BMI, maternal race and APGAR score at 5 minutes

** Adjusted for age at assessment, maternal age, gestational age at birth, smoking in pregnancy, income level, maternal BMI and APGAR score at 5 minutes

*** Adjusted for age at assessment, maternal age, smoking in pregnancy, income level, maternal BMI and APGAR score at 5 minutes

**** Adjusted for Age at assessment, maternal age, gestational age at birth, smoking in pregnancy, income level, maternal BMI

BW = Birth weight, HC = Head Circumference, POBW = Proportion of optimal birth weight, HCAC = Head-to-abdominal circumference, PI = Ponderal index, TRF = Teacher report form.

Supplementary table 6 Checking the specificity of the models by examining externalising scores in linear mixed models.

| Fetal growth parameter | Male parsimonious model. Fetal growth parameter | Female parsimonious model. Age interaction | Female parsimonious model. Fetal growth parameter |
| --- | --- | --- | --- |
| BW* | N = 1092  β: -0.444  95%CI: [-0.887, 0.0239] | NA | N = 971  β: 0.134  95%CI: [-0.330, 0.614] |
| HC** | N = 1073  β: -1.08  95%CI: [-1.53, -0.606] | N = 956  β: 0.0380  95%CI: [-0.0123, 0.0904] | N = 956  β: -0.652  95%CI: [-1.39, 0.0433] |
| POBW*** | N = 1082  *1. Order*  β: -0.251  95%CI: [-0.590, 0.0971]  *2. Order*  β: 0.109  95%CI: [-0.0977, 0.307] | NA | N = 969  *1. Order*  β: 0.262  95%CI: [-0.0580, 0.599]  *2. Order*  β: -0.0931  95%CI: [-0.313, 0.145] |
| HCAC**** | N = 1027  *1. Order*  β: -0.224  95%CI: [-0.572, 0.110]  *2. Order*  β: 0.160  95%CI: [-0.0588, 0.381] | NA | N = 916  *1. Order*  β: -0.147  95%CI: [-0.459, 0.142]  *2. Order*  β: -0.0760  95%CI: [-0.278, 0.121] |
| PI**** | N = 1073  β: -0.0256  95%CI: [-0.372, 0.331] | NA | N = 958  β: -0.0325  95%CI: [-0.330, 0.286] |

Supplementary table 6: Using externalising raw scores as mixed model outcome to test the specificity of relationship

* Adjusted for age at assessment, maternal age, gestational age at birth, smoking in pregnancy, income level, maternal BMI, maternal race and APGAR score at 5 minutes

** Adjusted for age at assessment, maternal age, gestational age at birth, smoking in pregnancy, income level, maternal BMI and APGAR score at 5 minutes

*** Adjusted for age at assessment, maternal age, smoking in pregnancy, income level, maternal BMI and APGAR score at 5 minutes

**** Adjusted for Age at assessment, maternal age, gestational age at birth, smoking in pregnancy, income level, maternal BMI

BW = Birth weight, HC = Head Circumference, POBW = Proportion of optimal birth weight, HCAC = Head-to-abdominal circumference, PI = Ponderal index.

| Parsimonious model Excluding 1 outlier n=1026 | Head circumference estimate | 1/Abdominal circumference estimate | Head-to-abdominal circumference estimate | Model fit: |
| --- | --- | --- | --- | --- |
| HC/AC + (HC/AC)^2 | NA | NA | *1. Order*  β: 0.00134  95%CI: [-0.145, 0.145]  *2. Order*  β: **0.170** 95%CI: [0.0718, 0.273] | AIC: 19130 |
| HC + HC^2 + 1/AC + (1/AC)^2 | *1. Order*  β: -0.202  95%CI: [-0.422, 0.00162]  *2. Order*  β: 0.0585 95%CI: [-0.0722, 0.188] | *1. Order*  β: 0.123  95%CI: [-0.0663, 0.309]  *2. Order*  β: 0.0680 95%CI: [ -0.0346 0.169] | NA | AIC: 19133 |
| HC+HC^2 + 1/AC+ (1/AC)^2 + HC/AC + (HC/AC)^2 | *1. Order*  β: -0.190  95%CI: [-18.2, 18.2]  *2. Order*  β: 0.106 95%CI: [-0.402, 0.613] | *1. Order*  β: 0.158  95%CI: [-27.9, 28.9]  *2. Order*  β: -0.0601 95%CI: [-1.21, 1.05] | *1. Order*  β: -0.0796  95%CI: [-22.4, 21.7]  *2. Order*  β: **0.190** 95%CI: [-0.466, 0.880] | AIC: 19131 |

Supplementary Table 7: Exploring the head-to-abdominal circumference ratio with fixed terms of HC and 1/AC

Supplementary table 7: Combinations of HC and 1/AC were included in models to test if it was individual components of the HC/AC or the ratio itself that drove associations with CBCL-AP. HC/AC-ratio outperformed models that included HC and 1/AC as individual terms. Additionally a model with all terms had minimal impact on the quadratic effect estimate of HC/AC (in **bold**)

All models adjusted for Age at assessment, maternal age, gestational age at birth, smoking in pregnancy, income level, maternal BMI

HC = Head Circumference, AC = Abdominal circumference, HCAC = Head-to-abdominal circumference, AC = abdominal circumference, CBCL-AP: Child Behaviour Checklist - Attention Problems.
